# Facile Preparation of the Tosylhydrazone Derivatives of a Series of Racemic *trans*-3,4-Substituted Cyclopentanones

**DOI:** 10.3390/molecules17010001

**Published:** 2011-12-22

**Authors:** Kamal H. Bouhadir, Bilal Abou Aleiwe, Fares A. Fares

**Affiliations:** 1 Department of Chemistry, American University of Beirut, Beirut 11-0236, Lebanon; 2 Department of Chemistry, Lebanese University, Hadath 6573-14, Lebanon

**Keywords:** tosyl hydrazone, cyclopentanone, ethylene ketal, monohydrolysis

## Abstract

We report the synthesis and characterization of a variety of *trans*-3,4-substituted cyclopentanones and the corresponding tosylhydrazone derivatives starting with diethyl fumarate. Protection of the keto group followed by selective monohydrolysis of esters was achieved, resulting in cyclopentanones with different substituents at positions 3 and 4. The tosylhydrazone derivative of each cyclopentanone intermediate was prepared in moderate to good yields. These compounds are potential precursors for functionalized methanofullerenes.

## 1. Introduction

Over the past two decades there has been an increasing interest in functionalized methanofullerenes [1,2]. These molecules have been utilized to couple proteins, oligonucleotides [3,4], and other macromolecules as well [5]. The reason behind this interest is the potential utility of this class of compounds in biomedical applications [6]. Fullerene derivatives such as fulleropyrrolidines are active against HIV-1 and HIV-2 and amino acid derivatives of fullerenes inhibit HIV and human cytomegalovirus replication [7,8]. In addition, water-soluble photoactive fullerenes have been investigated as potential photosensitizers for photodynamic therapy [9]. Several synthetic routes leading to methanofullerenes have been well established over the last two decades including the thermal addition of diazocompounds and carbenes to C_60_ [10]. We have previously reported the addition of tosylhydrazone salts to C_60_ resulting in the formation of methanofullerenes [11]. The tosylhydrazone derivative of 3,4-substituted cyclopentanone is an interesting entry to the formation of fuctionalized methanofullerenes and carbon nanotubes as well. We report herein the facile preparation of a series of *trans*-3,4-substituted cyclopentanones and the corresponding tosylhydrazone derivatives as potential adducts for the preparation of methanofullerenes.

## 2. Results and Discussion

**Scheme 1:**
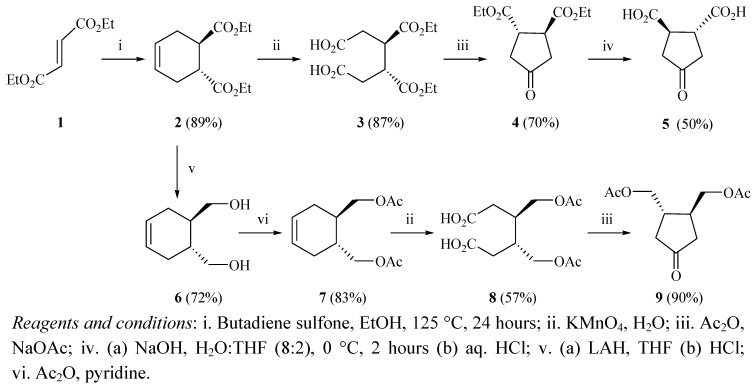
Preparation of intermediates **5** and **9**.

The first cyclopentanone derivative, *rac-trans*-3,4-bis(carboethoxy)cyclopentanone (**±**)**4** was synthesized following a reported procedure (Scheme 1) [12]. The Diels-Alder reaction between butadiene sulfone and diethylfumarate (**1**) was utilized to form *rac-trans*-4,5-bis(carboethoxy)-cyclohexene [(±)**2**] in 89% yield [8]. Intermediate (±)**2** was oxidized with potassium permanganate to yield the *rac-trans*-3,4-bis(carboethoxy)hexanedioic acid [(±)**3**] in 87% yield. Compound (±)**3** was allowed to react continuously with acetic anhydride (thus forming the mixed anhydride) followed by sodium acetate whereby it underwent a Dieckmann condensation to form *rac-trans*-3,4-bis(carboethoxy)cyclopentanone [(±)**4**] in 70% yield. We attempted at this point to selectively hydrolyze one of the ester groups in compound (±)**4** under alkaline conditions utilizing sodium hydroxide in aqueous tetrahydrofuran (20% v/v THF:H_2_O) at 0 °C [11]. However, we either isolated the starting diester (±)**4**, diacid (±)**5**, or a mixture of both. In contrast, stirring compound (±)**4** for two hours under these conditions formed racemic *trans*-3,4-bis(carboxy)cyclopentanone [(±)**5**] in 70% yield [13,14,15]. The desired monohydrolysis product was procured following a different route, as shown later on in this paper (Scheme 3).

The synthesis of the second cyclopentanone derivative was initiated with the reduction of compound (±)**2** in a stirred suspension of LAH in dry THF to form the racemic *trans*-4,5-bis(hydroxy-methyl)cyclohexene [(±)**6**] in 72% yield (Scheme 1) [21]. Compound (±)**6** was then acylated with acetic anhydride to form racemic *trans*-4,5-bis(acetoxymethyl)cyclohexene [(±)**7**] in 83% yield. Intermediate (±)**7** was oxidized with potassium permanganate to form racemic *trans*-3,4-bis(acetoxy-methyl)hexanedioic acid [(±)**8**] in 57% yield. Following this, compound (±)**8** was allowed to react with acetic anhydride and sodium acetate to undergo the a Dieckmann condensation and form racemic *trans*-3,4-bis(acetoxymethyl)cyclopentanone [(±)**9**] in 90% yield (Scheme 1) [11].

**Scheme 2:**
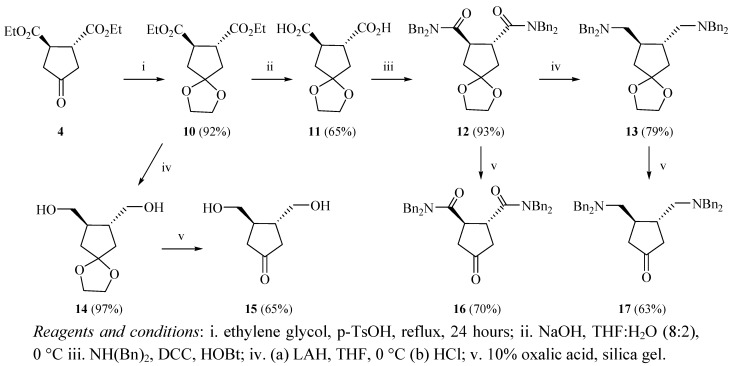
Preparation of intermediates **15**, **16** and **17**.

Intermediate **(±)4** was converted to the corresponding ethylene ketal (±)**10** in 92% yield (Scheme 2). Hydrolysis of the ester groups in compound (±)**10** was performed in alkaline aqueous tetrahydrofuran (80% v/v THF:H_2_O) at 0 °C to afford intermediate (±)**11** in 65% yield [16]. The free carboxylic acid groups were then coupled to dibenzylamine with dicyclohexylcarbodiimide (DCC) and *N*-hydroxybenzotriazole (HOBt) to form the corresponding amide (±)**12** in 93% yield. Reduction of intermediate (±)**12** in a stirred suspension of LAH in dry THF formed the ethylene ketal derivative of racemic *trans*-3,4-bis(*N*,*N*-dibenzylaminomethyl)cyclopentanone [(±)**13**] in 79% yield. In addition, compound (±)**10** was reduced with LAH to afford 3,4-bis(hydroxymethyl)cyclopentanone ethylene ketal [(±)**14**] in 97% yield. Deketalization of compounds (±)**12**, (±)**13**, and (±)**14** was accomplished by stirring each compound in an aqueous suspension of oxalic acid (10% w/v) over silica gel for 24 hours at room temperature to afford the corresponding compounds (±)**16**, (±)**17**, and (±)**15** in 70%, 63%, and 65% respectively (Scheme 2) [17].

**Scheme 3:**
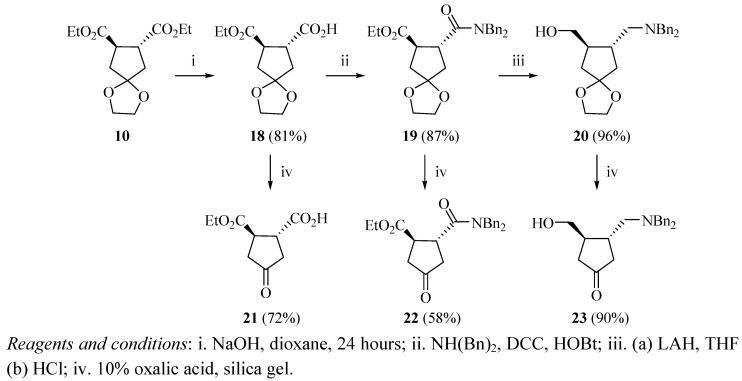
Preparation of compounds **21**, **22** and **23**.

The selective monohydrolysis of compound (±)**10** was achieved with sodium hydroxide in aqueous dioxane (50:50 H_2_O/dioxane) at room temperature to form intermediate (±)**18** in 81% yield (Scheme 3) [18]. The free carboxylic acid group of compound (±)**18** was coupled to dibenzylamine with DCC and HOBt to form the corresponding amide (±)**19** in 87% yield. Compound (±)**19** was then reduced with LAH to form the ethylene ketal intermediate (±)**20** in 96% yield. The ethylene ketal derivatives (±)**18**, (±)**19**, and (±)**20**, were separately hydrolyzed as before by stirring over 10% aqueous oxalic acid and silica gel for 24 hours at room temperature to afford compounds (±)**21**, (±)**22**, and (±)**23** in 72%, 58%, and 90% respectively (Scheme 3).

**Scheme 4:**
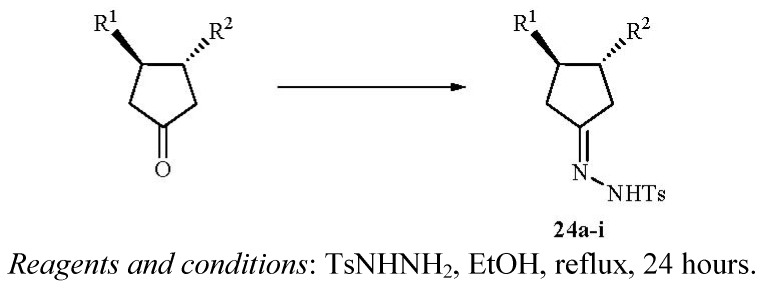
Preparation of the tosylhydrazone derivatives **24a–i**.

The tosylhydrazone derivative of each of the *trans*-3,4-substituted cyclopentanone intermediates was prepared following the classical method via the addition of each cyclopentanone derivative to a hot ethanolic solution of *p*-toluenesulfonyl hydrazide (Scheme 4) [19]. The detailed experimental protocol for the preparation of the reported derivative **24a** is included in this manuscript. The purified and isolated yield of each derivative varied between 46% for (±)**24h** (entry 8) and 81% for (±)**24b** (entry 2) as shown in Table 1.

**Table 1:** Experimental yields of the tosyl hydrazone derivatives **24a–i** as shown in Scheme 4.

Entry	R^1^	R^2^	Yield (%) ^a^	M.p. (°C)
1	-CO_2_CH_2_CH_3_	-CO_2_CH_2_CH_3_	**24a** (64)	163–164
2	-CH_2_OCOCH_3_	-CH_2_OCOCH_3_	**24b** (81)	ND ^c^
3	-CO_2_H	-CO_2_H	**24c** (59)	202–203 ^b^
4	-CH_2_OH	-CH_2_OH	**24d** (50)	ND ^c^
5	-CON(Bn)_2_	-CON(Bn)_2_	**24e** (54)	219–220
6	-CH_2_N(Bn)_2_	-CH_2_N(Bn)_2_	**24f** (52)	143–144
7	-CO_2_H	-CO_2_CH_2_CH_3_	**24g** (64)	199–200 ^b^
8	-CON(Bn)_2_	-CO_2_CH_2_CH_3_	**24h** (46)	177–178
9	-CH_2_N(Bn)_2_	-CH_2_OH	**24i** (66)	261–262 ^b^

^a^ Yields refer to isolated products; ^b^ Compound decomposed; ^c^ Not determined (hygroscopic).

## 3. Experimental

Melting points were determined on a Fisher-Johns apparatus and were uncorrected. NMR spectra were determined in deuterated solvents with TMS as the internal standard on a Bruker AM 300 NMR spectrometer. Chemical shifts are reported in ppm (δ) downfield relative to TMS. Infrared spectra were recorded as KBr pellets using a Nicolet AVATAR 360 FTIR ESP spectrometer with a Hewlett Packard Desk jet 840C plotter. The IR bands are reported in wavenumbers (cm^−1^). Thin layer chromatography (TLC) was performed on polygram Sil G/UV_254_ silica gel sheets. Preparative column chromatography employed ACROS silica gel (60 Å, 200–400 mesh). Preparative thin layer chromatography employed ALLTECH silica gel 60 F_254_ plates. Reagents used in the syntheses were purchased from the Aldrich Chemical Company (Milwaukee, WI, USA), ACROS Chemicals (Belgium), Fisher Scientific Company (Fair Lawn, NJ, USA), and were used as received.

*rac-trans-3,4-bis(Ethoxycarbonyl)cyclohexene* (**2**). A pressure vessel was charged with butadiene sulfone (60 g, 0.51 mol), diethyl fumarate (86 g, 82 mL, 0.5 mol), and hydroquinone (1 g, 9 mmol). Absolute ethanol (90 mL) was added and the mixture was stirred until most of the solid was dissolved. The vessel was sealed and heated slowly at 125 °C for 24 hours. The vessel was allowed to cool down to room temperature, and the yellowish mixture was transferred into an Erlenmeyer flask. The mixture was stirred vigorously and a solution of sodium carbonate (60 g, 0.57 mol) in water (350 mL) was slowly added. The mixture was stirred for an additional 20 minutes. Petroleum ether (200 mL) was added and the mixture was stirred for about 10 minutes at a rate sufficient to achieve homogenization. The organic layer was separated and the aqueous layer was extracted with petroleum ether (2 × 100 mL). The organic layers were combined and washed with aqueous sodium carbonate (5%, 100 mL) and with cold water (2 × 50 mL). The organic layer was dried over anhydrous MgSO_4_ and filtered. The solvent was removed under reduced pressure and the product was purified by vacuum distillation (115 °C, 4 mmHg) to yield an oil (99.5 g, 89%). ^1^H-NMR (CDCl_3_) δ 1.25 (t, 6H), 2.21 (m, 2H), 2.40 (m, 2H), 2.83 (m, 2H), 4.15 (q, 4H), 5.69 (t, 2H); ^13^C-NMR (CDCl_3_) δ 14.19 (q), 27.92 (t), 41.42 (d), 60.58 (t), 125.1 (d), 174.82 (s); FTIR (cm^−1^) 1,038.2, 1,073.2, 1,180.6, 1,037.5, 1,657.6, 1,735.4, 2,981.3, 2,937. The spectroscopic data were in good agreement with those reported in the literature [6].

*rac-trans-3,4-bis(Ethoxycarbonyl)hexanedioic acid* (**3**). A solution of potassium permanganate (75 g, 4.75 mmol) and water (365 mL) was stirred at room temperature for one hour. The mixture was cooled in an ice-water bath and a solution of *rac-trans*-3,4-bis(methoxycarbonyl)cyclohexene (**2**, 34 g, 150.3 mmol) in acetone (40 mL) was added drop-wise while maintaining the temperature below 10 °C. The reaction was stirred for 3 hours at room temperature. Sodium bisulfite was added (75 g) and the reaction was stirred for an extra 20 minutes. The reaction was then carefully acidified (pH 2) with concentrated HCl and the clear solution was extracted with EtOAc:THF 1:1 (2 × 200 mL). The organic layers were combined, dried over anhydrous MgSO_4_, and filtered. The solvent was evaporated under reduced pressure and the residue was crystallized from ethyl acetate and dried under reduced pressure to afford the product (38.2 g, 87%). Melting point: 170 °C (d); ^1^H-NMR (DMSO-d_6_) δ 1.16 (t, 6H), 2.38 (d, 2H), 2.44 (d, 2H), 3.02 (t, 2H), 12.18 (br s, 2H); ^13^C-NMR (DMSO-d_6_) δ 13.82 (q), 33.29 (t), 42.01 (d), 60.41 (t), 171.87 (s), 172.52 (s); FTIR (cm^−1^) 2,983.2, 1,728.7, 1,181.3, 1,235.8, 1,294.4, 1,019.5, 1,084.7. The spectroscopic data were in good agreement with those reported in the literature [17].

*rac-trans-3,4-bis(Ethoxycarbonyl)cyclopentanone* (**4**). *rac-cis*-3,4-bis(Ethoxycarbonyl)hexanedioic acid (**3**, 15 g, 51.7 mmol) and acetic anhydride (75 mL) mixed and heated at 130 °C for 1.5 hours. Sodium acetate (3.75 g) was added in one portion and stirred at 130 °C until carbon dioxide ceased to evolve (20 minutes). The flask was cooled down to room temperature and immersed in an ice-water bath. Methanol (75 mL) was carefully added to decompose the excess acetic anhydride followed by water (150 mL). Sodium bicarbonate (5 g) was carefully added portion-wise and the mixture was stirred in an ice-water bath until bubbles ceased to evolve. The solution was extracted with CH_−2_Cl_2_ (3 × 75 mL), and the combined organic layers were dried over anhydrous MgSO_4_ and filtered. The solvent was evaporated under reduced pressure and the product was purified by vacuum distillation (180 °C, 10 mmHg) to afford the product (9 g, 76%). ^1^H-NMR (CDCl_3_) δ 2.65 (m, 4H), 3.36 (m, 2H), 4.18 (q, 4H), 1.28 (t, 6H); ^13^C-NMR (CDCl_3_) δ 14.10 (q), 41.05 (t), 43.92 (d), 61.11 (t), 172.83 (s), 212.62 (s); FTIR (cm^−1^) 2,983.5, 1,735.8, 1,209.4, 1,033.2. The spectroscopic data were in good agreement with those reported in the literature [11].

*4-Oxocyclopentane-1,2-dicarboxylic acid* (**5**). *rac-trans*-3,4-bis(Ethoxycarbonyl)cyclopentanone (**4**, 0.8 g, 3.5 mmol) was dissolved in THF (7 mL) and water (50 mL) was added. The mixture was stirred for about one hour at room temperature. Sodium hydroxide (100 mL, 0.25 N) was added drop-wise during one hour at room temperature. The reaction was stirred for about 3 hours. The reaction was acidified to pH 2 using aqueous hydrochloric acid (1 N). The reaction was saturated with sodium chloride and the solution was extracted with ethyl acetate (3 × 50 mL). The combined organic layers were dried over anhydrous MgSO_4_ and filtered. The solvent was evaporated under reduced pressure to yield a solid (0.3 g, 50%). Melting point: 194–195 (d); ^1^H-NMR (DMSO-d_6_) δ 3.21 (m, 2H), 2.37–2.52 (m, 4H); ^13^C-NMR (DMSO-d_6_) δ 40.69 (t), 43.14 (d), 174.26 (s), 213.48 (s); FTIR (cm^−1^) 2,962.9 (br), 1,708.5, 1,282.4, 1,753.1. The spectroscopic data were in good agreement with those reported in the literature [20].

*rac-trans-4,5-bis(Hydroxymethyl)cyclohexene* (**6**). Lithium aluminum hydride (10 g, 0.27 mol) was added to dry THF (300 mL) under a nitrogen atmosphere at 0 °C. A solution of *rac-trans*-4,5-bis(ethoxycarbonyl)cyclohexene (**2**, 20 g, 88.5 mmol) in tetrahydrofuran (200 mL) was added drop-wise to the mixture. The mixture was stirred at room temperature for 1.5 hours then refluxed for 14 hours. The mixture was cooled to room temperature, and then dipped in an ice-water bath. Excess LAH was decomposed by the addition of water (19 mL). Tetrahydrofuran (400 mL) was added to the solution and then decomposition of LAH was continued by the addition of aqueous KOH (15% w/v, 19 mL), and water (19 mL). Stirring was continued for 10 minutes and the white solid was filtered and washed with diethyl ether (2 × 100 mL). The filtrate was dried over anhydrous MgSO_4_ and filtered. The solvent was removed under reduced pressure to yield the product (12 g, 72%): ^1^H-NMR (CDCl_3_) δ 1.68–1.86 (m, 4H), 1.99–2.04 (m, 2H), 5.62 (m, 2H), 3.54–3.74 (d, 4H), 4.71 (s, 2H); ^13^C-NMR (CDCl_3_) δ 27.98 (t), 39.01 (d), 65.94 (t), 125.91 (d); FTIR (cm^−1^) 3,353.2, 2,890.8, 1,655.3, 1,054.9.

*rac-trans-4,5-bis(Acetoxymethyl)cyclohexene* (**7**). *rac-trans*-4,5-bis(Hydroxymethyl)cyclohexene, (**6**, 3.69 g, 25.9 mmol) was added to a mixture of acetic anhydride (6.12 mL, 64.87 mmol) and sodium acetate (0.589 g, 7.2 mmol). The mixture was refluxed for 0.5 hours. The mixture was cooled down to room temperature followed by the addition of ice-cold water (30 mL) was added. The mixture was transferred to a separatory funnel and CH_2_Cl_2_ (40 mL) was added. The organic layer was separated and washed with an aqueous solution of sodium hydroxide (10% w/v, 2 × 25 mL). The organic layer was dried over anhydrous MgSO_4 _and filtered. The solvent was removed under reduced pressure to yield the product (4.89 g, 83%): ^1^H-NMR (CDCl_3_) δ 1.95–2.00 (m, 4H), 2.12–2.17 (m, 2H), 4.05–4.13 (m, 4H), 5.60 (s, 2H), 2.05 (s, 6H); ^13^C-NMR (CDCl_3_) δ 26.60 (t), 33.87 (d), 66.30 (t), 125.10 (d), 171.10 (s), 20.9 (q); FTIR (cm^−1^) 2,904.2, 1,739.6, 1,239.9, 1,366.8, 1,035.7, 1,658.2.

*rac-trans-3,4-bis(Acetoxymethyl)hexanedioic acid* (**8**). A solution of *rac-trans*-4,5-bis(acetoxymethyl)cyclohexene (**7**, 2.5 g, 11 mmol) in acetone (5 mL) was added dropwise to a solution of potassium permanganate (5 g, 33.19 mmol) and water (31 mL) while maintaining the temperature below 10 °C. The mixture was stirred at room temperature for 20 hours. Sodium metabisulfite (5.4 g, 28.4 mmol) was added and the mixture was stirred for 30 minutes. The solution was then carefully acidified (pH 2) with concentrated HCl. The clear solution was extracted with EtOAc-THF 1:1 (2 × 31 mL). The organic layers were combined, dried over anhydrous MgSO_4_, and filtered. The solvent was evaporated under reduced pressure to afford the product (2.73 g, 85%): ^1^H-NMR (DMSO-d_6_) δ 2.59–2.63 (d, 4H), 3.95–3.99 (m, 2H), 4.34–4.37 (m, 2H), 2.31–2.34 (t, 2H); ^13^C-NMR (DMSO-d_6_) δ 33.80 (t), 35.4 (d), 64.6 (t), 171.69 (s), 21.01 (q), 176.80 (s); FTIR (cm^−1^) 3,118.6, 1,732.8, 1,308.6, 1,247.8.

*rac-trans-3,4-bis(Acetoxymethyl)cyclopentanone* (**9**). *rac-trans*-3,4-bis(Acetoxymethyl)hexanedioic acid (**8**, 6 g, 20.7 mmol) and acetic anhydride (26 mL) were mixed and heated at 130 °C for 1.5 hours. Sodium acetate (1.694 g, 21 mmol) was added in one portion and the reaction was stirred at 130 °C until carbon dioxide ceased to evolve (1 hour). The flask was cooled to room temperature and immersed in an ice-water bath. Methanol (30 mL) was carefully added to decompose the excess acetic anhydride. Water (60 mL) was then added to the solution. Sodium bicarbonate (21.34 g, 254 mmol) was added portion-wise and the mixture was stirred in an ice-water bath until the bubbling ceased to evolve. The solution was extracted with CH_­2_Cl_2_ (2 × 34 mL) and the combined organic layers were dried over anhydrous MgSO_4_, filtered, and the solvent was evaporated under reduced pressure to afford the product (4.22 g, 90%): ^1^H-NMR (CDCl_3_) δ 2.16 (d, 4H), 2.43–2.48 (m, 2H), 4.17 (d, 4H), 2.048 (s, 6H); ^13^C-NMR (CDCl_3_) δ 41.49 (t), 37.82 (d), 170.91 (s), 20.68 (q), 215.85 (s); FTIR (cm^−1^) 1,739, 1,035.7, 1,239.7, 1,308.6.

*rac-trans-3,4-bis(Ethoxycarbonyl)cyclopentanone ethylene ketal* (**10**). *rac-trans*-3,4-bis(Ethoxy-carbonyl)cyclopentanone (**4**, 6 g, 26.3 mmol) was added to a solution of ethylene glycol (60 mL) and *p*-toluenesulfonic acid monohydrate (0.2 g) in toluene (270 mL). The mixture was refluxed for 20 hours. Sodium bicarbonate (0.12 g) was added and stirring was continued for 5 minutes. The reaction mixture was washed with a saturated solution of sodium bicarbonate (90 mL). The solution was extracted with CH_2_Cl_2_ (3 × 60 mL) and the organic layers were combined, dried over anhydrous MgSO_4_, and filtered. The solvent was removed under reduced pressure to yield an oil (6.6 g, 92%). ^1^H-NMR (CDCl_3_) δ 1.25 (t, 6H), 4.13 (q, 4H), 3.1 (m, 2H), 2.07 (m, 2H), 2.22 (m, 2H), 3.90 (m, 4H); ^13^C-NMR (CDCl_3_) δ 14.19 (q), 60.87 (t), 44.43 (d), 39.21 (t), 64.58 (t), 115.52 (s), 173.8 (s); FTIR (cm^−1^) 2,982.4, 1,735.8, 1,179.6, 1,325.5, 1,377.2, 1,036.4, 1,066.

*4-Oxocyclopentane-1,2-dicarboxylic acid ethylene ketal* (**11**). *rac-trans*-3,4-bis(Ethoxycarbonyl)cyclopentanone ethylene ketal (**10**, 1.1 g, 4 mmol) was dissolved in THF (10 mL) and water (80 mL). The reaction mixture was immersed in an ice-water bath and cooled down to 0 °C. Aqueous sodium hydroxide (100 mL, 0.25 N) was added drop-wise with stirring during one hour and the reaction was stirred at the same temperature for an additional hour, and then acidified with aqueous HCl (1 N). The reaction mixture was saturated with NaCl and the product was extracted with ethyl acetate (3 × 60 mL). The combined organic layers were dried over anhydrous MgSO_4_ and filtered. The solvent was evaporated under reduced pressure to yield a solid (0.56 g, 65%). ^1^H-NMR (DMSO-d_6_) δ 1.19 (m, 2H), 2.14 (m, 2H), 3.01 (m, 2H), 3.82 (s, 4H); ^13^C-NMR (DMSO-d_6_) δ 39.08 (t), 43.61 (d), 63.77 (t), 114.88 (s), 174.66 (s).

*4-Oxo-cyclopentane-1,2-dicarboxylic acid bis-dibenzylamide ethylene ketal* (**12**). Dicyclohexyl-carbodiimide (4 g, 19.38 mmol) was added to a stirred solution of HOBT (1 g, 8 mmol) and 4-oxo-cyclopentane-1,2-dicarboxylic acid **11** (1 g, 5.81 mmol) in DMF (50 mL) at 0 °C and stirred for 90 minutes at room temperature. The urea was filtered off and the filtrates were added to a solution of dibenzyl amine (1.97 g, mmol) in DMF (50 mL). The mixture was stirred overnight and the solvent was removed under reduced pressure. The solid residue was dissolved in ethyl acetate and washed with a saturated solution of NaHCO_3_. The mixture was dried over anhydrous MgSO_4_ and filtered. The solvent was evaporated under reduced pressure to yield an oil (2.8 g, 93%). ^1^H-NMR (CDCl_3_) δ 2.3–2.4 (m, 4H), 3.80–3.83 (m, 2H), 4.1–4.13 (m, 4H), 4.30–4.36 (m, 4H), 7.14–7.34 (m, 20H); ^13^C-NMR (CDCl_3_) δ 42.48 (d), 42.82 (t), 48.93 (t), 49.13 (t), 126.41 (d), 127.52 (d), 127.83 (d), 128.16 (d), 128.41 (d), 129.05 (d), 136.28 (s), 136.85 (s), 173.39 (s), 213.15 (s). FTIR (cm^−1^) 3,387, 3,260, 1,635.3, 1,310, 1,157.33, 1,090.6, 815.4, 698.4, 551.8.

*3,4-bis-(Dibenzylaminomethyl)cyclopentanone ethylene ketal* (**13**). Lithium aluminum hydride (0.3 g, 95%, 7.9 mmol) was added to dry THF (8 mL) under a nitrogen atmosphere. A solution of compound **12** (0.7 g, 1.22 mmol) in dry THF (6 mL) was added to the mixture dropwise. The mixture was refluxed overnight then cooled down to 0 °C in an ice-water bath. The reaction was carefully quenched with water (10 mL), aqueous KOH (15% w/v, 6 mL), and water (6 mL). The solution was filtered and the solid washed with diethyl ether. The combined filtrate and organic washes were dried over anhydrous MgSO_4_ and filtered. The solvent was evaporated under reduced pressure to form an oily residue that was triturated with hexane to yield a solid (0.52 g, 78%). ^1^H-NMR (CDCl_3_) δ 1.22–1.26 (m, 2H), 1.53 (m, 4H), 1.93 (m, 2H), 2.33 (m, 2H), 3.28–3.33 (m, 8H), 3.68–3.76 (m, 4H), 7.21–7.33 (m, 20H); ^13^C-NMR (CDCl_3_) δ 30.93 (t), 39.39 (t), 40.96 (d), 58.59 (t), 59.06 (t), 63.92 (t), 116.47 (s), 126.77 (d), 128.12 (d), 128.87 (d), 139.71 (s).

*The ethylene ketal of rac-trans-3,4-bis(hydroxymethyl)cyclopentanone* (**14**). Lithium aluminum hydride (5.88 g, 147.05 mmol) was added to dry THF (180 mL) under a nitrogen atmosphere at 0 °C. A mixture of *rac-trans*-3,4-bis(ethoxycarbonyl)cyclopentanone ethylene ketal (10 g, 36.76 mmol) and tetrahydrofuran (115 mL) was added dropwise and the mixture was stirred at room temperature for 0.5 hours and refluxed overnight. The reaction mixture was cooled to 0 °C using an ice-water bath. Excess LAH was decomposed by successive additions of water (12 mL), aqueous KOH (15% w/v 12 mL), and water (12 mL). Stirring was continued for 10 minutes and the white solid was filtered and washed with diethyl ether (2 × 50 mL). The combined washes and filtrates were dried over anhydrous MgSO_4_, and filtered. The solvent was removed under reduced pressure to yield the product (6.7 g, 97%): ^1^H-NMR (CDCl_3_) δ 3.90 (s, 4H), 1.95–2.01 (d, 4H), 1.47–1.56 (q, 2H), 3.43–3.49 (m, 2H), 3.70–3.73 (m, 2H), 4.79 (s, 2H); ^13^C-NMR (CDCl_3_) δ 39.12 (t), 44.67 (d), 64.12 (t), 65.61 (t), 115.58 (s); FTIR (cm^−1^) 3,384.8, 2,888.5, 1,012.1, 1,084.7, 949.4.

*rac-trans-3,4-bis(Hydroxymethyl)cyclopentanone* (**15**). An aqueous solution of oxalic acid (10% w/v, 12.75 mL, 55 drops) was added to a stirred suspension of silica gel (12 g) and methylene chloride (16 mL). After adsorption of the aqueous phase on the silica gel surface, rac-trans-3,4-bis(hydroxymethyl)cyclopentanone ethylene ketal (4 g, 21.2 mmol) was added and the mixture was stirred for 3 days. Sodium bicarbonate (0.5 g, 5.95 mmol) was added to neutralize the mixture and the silica gel was filtered out and washed with ethyl acetate (70 mL). The organic washes were combined and the solvent was removed under reduced pressure to yield an oil (2 g, 65.36%): ^1^H-NMR (CDCl_3_) δ 1.99–2.46 (m, 4H), 2.88–2.98 (m, 4H), 3.55–3.6 (m, 2H), 3.69–3.82 (m, 2H), 4.74 (s, 2H); ^13^C-NMR (CDCl_3_) δ 41.92 (t), 43.98 (d), 64.95 (t), 217.10 (s); FTIR (cm^−1^) 1,734.9, 3,371.1, 2,931.8, 1,048.4.

*4-Oxocyclopentane-1,2-dicarboxylic acid bis-dibenzylamide* (**16**). An aqueous solution of oxalic acid (10% w/v, 20 drops) was added to a stirred suspension of silica gel (3 g) and CH_2_Cl_2_ (20 mL). A solution of compound **12** (0.5 g, 0.87 mmol) in dichloromethane (10 mL) was added and the mixture was stirred for one day. Sodium bicarbonate (0.5 g) was added to neutralize the mixture. The mixture was filtered and the silica gel was washed with ethyl acetate (2 × 20 mL) to remove any adsorbed product. The organic layers were combined and the solvent was removed under reduced pressure to yield an oil (0.32 g, 70%). ^1^H-NMR (CDCl_3_) δ 2.3–2.4 (m, 4H), 3.80–3.83 (m, 2H), 4.1–4.13 (m, 4H), 4.30–4.36 (m, 4H), 7.14–7.34 (m, 20H); ^13^C-NMR (CDCl_3_) δ 42.48 (d), 42.82 (d), 48.93 (t), 49.13 (t), 126.41 (d), 127.52 (d), 127.83 (d), 128.16 (d), 128.41 (d), 129.05 (d), 136.28 (s), 136.85 (s), 173.39 (s), 213.15 (s).

*3,4-bis-(Dibenzylaminomethyl)cyclopentanone* (**17**). An aqueous solution of oxalic acid (10% w/v, 20 drops) was added to a stirred suspension of silica gel (5 g) and CH_2_Cl_2_ (20 mL). A solution of compound **13** (1 g, 1.83 mmol) in dichloromethane (10 mL) was added and the mixture was stirred for one day. Sodium bicarbonate (0.5 g) was added to neutralize the mixture. The mixture was filtered and the silica gel was washed with ethyl acetate (2 × 20 mL) to remove any adsorbed product. The organic layers were combined and the solvent was removed under reduced pressure to yield an oily product (0.58 g, 63%). ^1^H-NMR (CDCl_3_) δ 0.91 (m, 2H), 1.79–1.83 (m, 4H), 2.18 (m, 4H), 3.38 (d, 4H), 3.59 (d, 4H), 7.21–7.35 (m, 20H); ^13^C-NMR (CDCl_3_) δ 28.90 (t), 37.84 (d), 43.11(d), 57.87 (t), 58.85 (t), 127.2 (d), 128.26 (d), 129.21 (d), 139.31 (s), 219.27 (s).

*4-Oxocyclopentane-1,2-dicarboxylic acid monoethyl ester ethylene ketal* (**18**). *rac-trans*-3,4-bis(Ethoxycarbonyl)cyclopentanone ethylene ketal (**10**, 1.638 g, 6 mmol) was dissolved in water/dioxane 1:1 (30 mL). After stirring for 10 minutes at room temperature aqueous, sodium hydroxide (50 mL, 0.25 N) was added drop-wise during one hour. The reaction mixture was then stirred for an additional hour at room temperature. The solvent was evaporated under reduced pressure and the residue was dissolved in water (30 mL). The aqueous layer was washed with chloroform (2 × 30 mL). The aqueous layer was acidified (pH 2) with aqueous HCl (1 N). The solution was extracted with chloroform (3 × 50 mL) and the organic layer was evaporated under reduced pressure to give 1.2 g of an oily product (81%). ^1^H-NMR (CDCl_3_) δ 1.25 (t, 3H), 2.11 (dd, 2H), 2.21 (dd, 2H), 3.23 (dt, 2H), 3.91 (s, 4H), 4.17 (q, 2H); ^13^C-NMR (CDCl_3_) δ 14.12 (q), 38.64 (t), 39.11 (t), 43.91 (d), 44.37 (d), 60.99 (t), 64.60 (t), 115.44 (s), 173.71 (s), 178.63 (s); FTIR (cm^−1^) 3,500, 3,202, 1,715.6 (br), 1,406, 1,341, 1,193.8, 1,156.8, 1,024.9, 547.7.

*2-Dibenzylcarbamoyl-4-oxo-cyclopentanecarboxylic acid ethyl ester ethylene ketal* (**19**). Dicyclohexylcarbodiimide (0.1 g, 0.4 mmol) was added to a stirred solution of HOBt (0.054 g, 0.4 mmol) and 4-oxocyclopentane-1,2-dicarboxylic acid monoethyl ester ethylene ketal (**18**, 0.1 g, 0.4 mmol) in dry DMF (2 mL) at 0 °C and stirred for 90 minutes. After stirring 30 minutes at room temperature DCU was filtered and the filtrate was added to a solution of dibenzyl amine (0.0786 g, 0.4 mmol) in dry DMF (2 mL). After stirring overnight the solvent was evaporated under reduced pressure. The crude was dissolved in ethyl acetate (10 mL) and washed with saturated solution of NaHCO_3_ and brine. The organic layer was washed with aqueous HCl (1 N, 10 mL) followed by aqueous NaOH (5% w/v, 10 mL). The organic layer was dried over anhydrous MgSO_4_ and filtered. The solvent was evaporated under reduced pressure to give the product which was loaded on a silica gel column chromatography and eluted first with methylene chloride isolating the excess DCU and then with ethyl acetate to yield an oil after the evaporation of the solvent (0.15 g, 86%). ^1^H-NMR (CDCl_3_) δ 1.22 (t, 3H), 4.16 (q, 2H), 2.01 (m, 2H), 2.25 (m, 2H), 3.63 (m, 2H), 4.15–4.62 (m, 4H), 7.19–7.66 (m, 10H); ^13^C-NMR (CDCl_3_) δ 14.20 (q), 39.17 (t), 38.71 (t), 41.90 (d), 44.33 (d), 48.33 (t), 49.84 (t), 60.86 (t), 64.55 (t), 115.68 (s), 126.20 (d), 126.74 (d), 127.34 (d), 127.60 (d), 127.66 (d), 128.03 (d),128.59 (d), 128.70 (d), 128.75 (d), 128.10 (d), 136.52 (s), 137.22 (s), 173.88 (s), 173.93 (s); FTIR (cm^−1^) 3,442.7, 3,029.3, 2,979.9, 1,728.7, 1,644.7, 1,495.7, 1,451.7, 1,187.1, 1,030.8.

*3-Dibenzylaminomethyl-4-hydroxymethylcyclopentanone ethylene ketal* (**20**). Lithium aluminum hydride (0.4 g, 95%, 10.54 mmol) was added to dry THF (25 mL) under a nitrogen atmosphere at 0 °C. A solution of compound **19** (0.6 g 1.418 mmol) in dry THF (25 mL) was added drop-wise and the mixture was stirred for 30 minutes. The reaction was refluxed overnight then cooled down in an ice-water bath .The reaction was carefully quenched with water (10 mL), aqueous KOH (10% w/v, 6 mL) and water (6 mL). The solution was filtered and the solid washed with diethyl ether. The combined organic washes were dried over anhydrous MgSO_4_ and filtered. The solvent was evaporated under reduced pressure to yield 0.5 g of an oily product (96%). ^1^H-NMR (CDCl_3_) δ 1.25–1.29 (m, 2H), 1.41–1.46 (m, 2H), 1.98 (m, 1H), 2.35 (m, 1H), 3.29–3.40 (m, 4H), 3.80 (m, 2H), 3.85 (m, 4H), 3.89 (m, 2H), 7.21–7.4 (m, 10H); ^13^C-NMR (CDCl_3_) δ 39.79 (t), 41.47 (d), 42.24 (t), 47.09 (d), 58.67 (t), 58.71 (t), 64.12 (t), 64.31 (t), 66.25 (t), 67.96 (t), 115.16 (s), 126.73 (d), 128.44 (d), 129.81 (d), 136.67 (s).

*4-Oxocyclopentane-1,2-dicarboxylic acid monoethyl ester* (**21**). An aqueous solution of oxalic acid (10% w/v, 20 drops) was added to a stirred suspension of silica gel (3 g) and CH_2_Cl_2_ (20 mL). A solution of compound **18** (1 g, 4.62 mmol) in dichloromethane (15 mL) was added and the mixture was stirred for one day. Sodium bicarbonate (1 g) was added to neutralize the mixture and the mixture was filtered. The silica gel was washed with ethyl acetate (2 × 30 mL) to remove any adsorbed product. The organic layers were combined and the solvent was removed under reduced pressure to yield the product (0.57 g, 71%). ^1^H-NMR (CDCl_3_) δ 1.28 (t, 3H), 2.57 (m, 2H), 2.67 (m, 2H), 3.41 (m, 2H), 4.22 (q, 2H); ^13^C-NMR (CDCl_3_) δ 14.08 (q), 40.817 (t), 40.95 (t), 43.50 (d), 43.62 (d), 61.74 (t), 172.80 (s), 177.57 (s), 213.01 (s).

*2-Dibenzylcarbamoyl-4-oxo-cyclopentanecarboxylic acid ethyl ester* (**22**). An aqueous solution of oxalic acid (10% w/v, 20 drops) was added to a stirred suspension of silica gel (3 g) and CH_2_Cl_2_ (20 mL). A solution of compound **19** (1 g, 2.36 mmol) in dichloromethane (15 mL) was added and the mixture was stirred for one day. Sodium bicarbonate (1 g) was added to neutralize the mixture. The mixture was filtered and the silica gel was washed with ethyl acetate (2 × 30 mL) to remove any adsorbed product. The organic layers were combined and the solvent was removed under reduced pressure to yield an oily product (0.52 g, 58%). ^1^H-NMR (CDCl_3_) δ 1.25 (m, 3H), 2.71 (m, 2H), 2.38 (m, 2H), 3.62 (m, 2H), 4.13 (m, 2H), 4.37 (dd, 2H), 4.75 (dd, 2H), 7.14–7.36 (m, 10H); ^13^C-NMR (CDCl_3_) δ 14.13 (q), 40.56 (t), 40.91 (t), 42.61 (d), 44.37 (d), 48.97 (t), 50.05 (t), 61.38 (t), 126.24 (d), 127.58 (d), 127.64 (d), 127.85 (d), 128.06 (d), 128.69 (d), 129.07 (d), 136.29 (s), 136.91 (s), 173.09 (s), 173.46 (s), 213.06 (s); FTIR (cm^−1^) 3,442, 3,029.3, 2,979.9, 1,728.7, 1,643.7, 1,451.7, 1,187.1, 1,363.9, 1,187.1, 1,030.8, 701.2.

*3-(Dibenzylaminomethyl)-4-(hydroxymethyl)cyclopentanone* (**23**). An aqueous solution of oxalic acid (10% w/v, 20 drops) was added to a stirred suspension of silica gel (3 g) and CH_2_Cl_2_ (5 mL). A solution of compound **20** (0.5 g) in dichloromethane (10 mL) was added and the mixture was stirred for one day. Sodium bicarbonate (0.5 g) was added to neutralize the mixture. The mixture was filtered and the silica gel was washed with ethyl acetate (20 mL) to remove any adsorbed product. The organic layers were combined and the solvent was removed under reduced pressure to yield an oily product (0.4 g, 90%). ^1^H-NMR (DMSO-d_6_) δ 1.03–1.08 (m, 2H), 1.24–1.26 (m, 4H), 3.85 (m, 2H), 3.39–3.48 (m, 4H), 4.20 (m, 2H), 7.41–7.90 (m, 10H); ^13^C-NMR (DMSO-d_6_) δ 39.38 (t), 42.05 (t), 40.55 (d), 43.14 (d), 55.93 (t), 57.41 (t), 61.37 (t), 62.69 (t), 127.82 (d), 128.41 (d), 129.57 (d), 161.47 (s), 216.62 (s).

### 3.1. General Protocol for the Preparation of the Tosyl Hydrazone Derivatives ***24a–i***

p-Toluenesulfonyl hydrazide (1.02 g, 5.48 mmol) was dissolved in hot ethanol (13 mL). The desired cyclopentanone (4.38 mmol) was added followed by water (1 mL) and the hot mixture was allowed to stand at room temperature for 30 minutes. A white solid precipitated, filtered, and recrystallized from ethanol to yield the corresponding p-tosylhydrazone derivative **24a–i**.

*rac-trans-3,4-bis(Ethoxycarbonyl)cyclopentanone* *p-tosylhydrazone* (**24a**). ^1^H-NMR (DMSO-d_6_) δ 1.17 (t, 6H), 2.38 (s, 3H), 2.57 (m, 4H), 3.16–3.19 (m, 2H), 4.07 (q, 4H), 7.34 (d, 2H), 7.69 (d, 2H); ^13^C-NMR (DMSO-d_6_) δ 13.87 (q), 20.91 (q), 31.80 (t), 35.52 (t), 44.52 (d), 44.77 (d), 60.40 (t), 127.35 (d), 129.36 (d), 137.21 (s), 143.08 (s), 162.10 (s), 172.33 (s); FTIR (cm^−1^) 3,441.6, 3,219.2, 2,984.3, 1,733.9, 1,597.9, 1,475.7, 1,410.8, 1,338.6, 1,093.1, 1,028.5, 923.4.

*4-Oxocyclopentane-1,2-dicarboxylic acid* *p-tosylhydrazone* (**24c**). ^1^H-NMR (DMSO-d_6_) δ 2.38 (s, 3H), 2.94 (m, 1H), 3.06 (m, 1H), 2.56 (m, 2H), 2.73 (m, 2H), 7.37–7.40 (d, 2H), 7.70–7.73 (d, 2H), 10.14 (s, 1H); ^13^C-NMR (DMSO-d_6_) δ 20.91 (q), 31.69 (t), 35.56 (t), 44.59 (d), 44.71 (d), 127.35 (d), 129.37 (d), 136.21 (s), 143.1 (s), 162.96 (s), 174.04 (s), 174.19 (s); FTIR (cm^−1^) 3,200.3, 2,923.9, 1,701.7, 1,598.2, 1,409, 1,349.2, 1,288.8, 1,162.01, 1,092.1, 1,021.5, 918.2.

*4-Oxocyclopentane-1,2-dicarboxylic acid bis-dibenzylamide* *p-tosylhydrazone* (**24e**). ^1^H-NMR (CDCl_3_) δ 2.40 (s, 3H), 2.33 (d, 2H), 2.63 (d, 2H), 3.64–3.80 (m, 2H), 4.35–4.91 (m, 8H), 7.10–7.32 (m, 22H), 7.74 (d, 2H); ^13^C-NMR (CDCl_3_) δ 21.61 (q), 32.31 (t), 37.71 (t), 43.63 (d), 44.33 (d), 48.51 (t), 49.24 (t), 50.45 (t), 50.49 (t), 126.41(d), 126.57(d), 127.46(d), 127.81(d), 127.90 (d), 127.93 (d), 128.72 (d), 129.00 (d), 129.10 (d), 129.68 (d), 129.67 (d), 136 (s), 136.49 (s), 136.75 (s), 136.83 (s), 144.23 (s), 161.70 (s), 172.72 (s), 173.22 (s); FTIR (cm^−1^) 3,387.7, 3,258.5, 2,926, 1,633.9, 1,598.4, 1,494.6, 1,450.4, 1,307.1, 1,156.2, 1,307.1, 1,156.2, 815.4.

*3,4-bis-(Dibenzylaminomethyl)cyclopentanone* *p-tosylhydrazone* (**24f**). ^1^H-NMR (DMSO-d_6_) δ 1.85 (m, 2H), 2.28 (m, 4H), 2.37 (s, 3H), 3.55 (m, 8H), 4.45 (m, 4H), 7.23–7.39 (m, 20H), 7.55 (d, 2H), 7.70 (d, 2H); ^13^C-NMR (DMSO-d_6_) δ 39.36 (t), 39.92 (d), 39.64 (d), 57.61 (t), 57.76 (t), 124.38 (d), 125.38 (d), 126.97 (d), 127.27 (d), 127.92 (d), 128.13 (d), 128.60 (d), 129.33 (d), 136.40 (s), 141.17 (s), 142.94 (s), 164.65 (s); FTIR (cm^−1^) 3,378.6, 3,022.7, 2,960.6, 2,812, 1,597.5, 1,493.2, 1,453.8, 1,337.9, 1,165, 1,086.1, 1,032.2, 967.6.

*4-Oxocyclopentane-1,2-dicarboxylic acid monoethyl ester* *p-tosylhydrazone* (**24g**). ^1^H-NMR (DMSO-d_6_) δ 1.15 (t, 2H), 2.37 (s, 3H), 2.42 (m, 2H), 2.81 (m, 2H), 2.91 (m, 1H), 3.21 (m, 1H), 4.07 (q, 2H), 7.09 (d, 2H), 7.67 (d, 2H), 10.17 (s, 1H); ^13^C-NMR (DMSO-d_6_) δ 13.85 (q), 20.91 (q), 30.59 (t), 31.66 (t), 44.85 (d), 44.62 (d), 60.33 (t), 127.35 (d), 129.37 (d), 136.18 (s), 143.11 (s), 162.50 (s), 172.59 (s), 173.76 (s); FTIR (cm^−1^) 3,200.3, 2,923.9, 1,701.9, 1,598.2, 1,409, 1,349.2, 1,288.8, 1,162, 1,092.1, 1,021.5, 918.2.

*2-Dibenzylcarbamoyl-4-oxo-cyclopentanecarboxylic acid ethyl ester* *p-tosylhydrazone* (**24h**). ^1^H-NMR (DMSO-d_6_) δ: 1.21 (m, 3H), 2.42 (s, 3H), 2.61–2.90 (m, 4H), 3.40 (m, 1H), 3.65 (m, 1H), 4.12 (q, 2H), 4.60–4.95 (dd, 2H), 4.36–4.60 (dd, 2H), 7.12–7.37 (m, 12H), 7.78–7.81 (m, 2H); ^13^C-NMR (DMSO-d_6_) δ: 13.86 (q), 20.92 (q), 37.13 (t), 39.51 (d), 41.46 (t), 45.23 (d), 48.12 (t), 49.66 (t), 60.51 (t), 126.50–129.40 (d), 136.17–137.21 (s), 143.20 (s), 162.64 (s), 172.52 (s), 172.71 (s); FTIR (cm^−1^) 3,200.5, 1,734.2, 1,635, 1,410.9, 1,338.6, 1,165.2, 1,093.9, 1,029.4.

*3-(Dibenzylaminomethyl)-4-(hydroxymethyl)cyclopentanone* *p-tosylhydrazone* (**24i**). ^1^H-NMR (DMSO-d_6_) δ 2.28 (m, 4H), 2.38 (s, 3H), 2.51 (m, 2H), 3.45 (m, 2H), 3.99–4.21 (m, 4H), 4.18 (m, 2H), 7.32–7.42 (m, 10H), 7.62–7.70 (m, 4H); ^13^C-NMR (DMSO-d_6_) δ 20.91 (q), 39.91 (d), 40.19 (d), 38.52 (t), 49.88 (t), 62.21 (t), 127.44 (d), 127.53 (d), 128.10 (d), 128.52 (d), 128.75 (d), 129.19 (d), 129.23 (d), 129.86 (d), 136.14 (s), 143.26 (s), 161.45 (s); FTIR (cm^−1^) 3,624.9, 3,303.6, 2,822.1, 1,683.6, 1,519, 1,445.1, 1,336.5, 1,159.1, 1,093.9, 1,048.8, 1,019.8, 856.6.

## 4. Conclusions

The aim of this work was to synthesize a series of racemic *trans*-3,4-substituted cyclopentanones and prepare the corresponding tosylhydrazone derivatives. The key intermediate for most of these molecules was the known *trans*-3,4-(diethoxycarbonyl)cyclopentanone. The tosylhydrazone derivatives were prepared as potential precursors for functionalized methanofullerenes. 
